# A web-based Alcohol Clinical Training (ACT) curriculum: Is in-person faculty development necessary to affect teaching?

**DOI:** 10.1186/1472-6920-8-11

**Published:** 2008-03-06

**Authors:** Daniel P Alford, Jessica M Richardson, Sheila E Chapman, Catherine E Dubé, Robert W Schadt, Richard Saitz

**Affiliations:** 1 Clinical Addiction Research and Education (CARE) Unit, Section of General Internal Medicine, Department of Medicine, Boston Medical Center, Boston, MA, USA; 2 Boston University School of Medicine, Boston, MA, USA; 3 Department of Community Health, Brown University, Providence, RI, USA; 4 Department of Educational Technology, Boston University School of Public Health, Boston, MA, USA; 5 Youth Alcohol Prevention Center, Boston University School of Public Health, Boston, MA, USA; 6 Department of Epidemiology, Boston University School of Public Health, Boston, MA, USA

## Abstract

**Background:**

Physicians receive little education about unhealthy alcohol use and as a result patients often do not receive efficacious interventions. The objective of this study is to evaluate whether a free web-based alcohol curriculum would be used by physician educators and whether in-person faculty development would increase its use, confidence in teaching and teaching itself.

**Methods:**

Subjects were physician educators who applied to attend a workshop on the use of a web-based curriculum about alcohol screening and brief intervention and cross-cultural efficacy. All physicians were provided the curriculum web address. Intervention subjects attended a 3-hour workshop including demonstration of the website, modeling of teaching, and development of a plan for using the curriculum. All subjects completed a survey prior to and 3 months after the workshop.

**Results:**

Of 20 intervention and 13 control subjects, 19 (95%) and 10 (77%), respectively, completed follow-up. Compared to controls, intervention subjects had greater increases in confidence in teaching alcohol screening, and in the frequency of two teaching practices – teaching about screening and eliciting patient health beliefs. Teaching confidence and teaching practices improved significantly in 9 of 10 comparisons for intervention, and in 0 comparisons for control subjects. At follow-up 79% of intervention but only 50% of control subjects reported using any part of the curriculum (p = 0.20).

**Conclusion:**

In-person training for physician educators on the use of a web-based alcohol curriculum can increase teaching confidence and practices. Although the web is frequently used for disemination, in-person training may be preferable to effect widespread teaching of clinical skills like alcohol screening and brief intervention.

## Background

Practice guidelines of leading professional societies recommend alcohol screening and behavioral counseling interventions in primary care settings [1-3]. Valid, brief, practical screening tools exist for the detection of unhealthy alcohol use in primary care settings[4], and brief interventions by physicians can reduce drinking and improve health outcomes when delivered to primary care patients with unhealthy alcohol use [5-8]. However, unhealthy alcohol use in primary care is often unrecognized and untreated, as reported in studies performed well after research demonstrating efficacy and national guidelines were published [9-14]. Although physicians recognize their responsibility in identifying and addressing alcohol problems [15], it often does not occur using effective patient-centered techniques [16]. Physician avoidance of and discomfort with brief alcohol counseling have been identified as important barriers [17].

Physician education can improve screening and brief intervention skills resulting in decreased patient drinking [7,18-22]. Some education and training programs aimed at improving physician attitudes and clinical practice around substance abuse issues have been effective [23-32]. However, despite the existence of numerous curricula [33], they are not being widely used [34]. Only half of internal medicine residency training programs have training on initial diagnosis and management of substance use disorders [34].

Web-based training can be an innovative and efficient way to connect with many individuals, while allowing learning at a convenient time for the learner. Adult learning principles [35] suggest that physicians' use of information sources outside the local sphere, such as journals, conferences, and the Internet, are essential to the enhancement and acceleration of information diffusion throughout the medical community. Although journals and books are the most common mechanism by which research findings are disseminated, they are not always read by practicing physicians [36]. The Internet can provide flexible, adaptable, tailored and sustainable access to current information [37-41] allowing for self-directed and individualized learning. Physicians have come to rely on the Internet for accessing clinical information [42] and for continuing medical education (CME) [43]. Internet-based CME has been shown to improve physician knowledge and change physician behavior [44-48]. However, little data is available on the use of web-based curricular materials by physician educators or the effectiveness of faculty development programs aimed at increasing physician use of the Internet curriculum resources. Further, although the train-the-trainer model is an efficient and widely accepted mechanism of curriculum dissemination, it is not known whether and to what degree such efforts enhance physician use of web-based curriculum tools.

To enhance dissemination of alcohol skills training to physicians, we developed an easily transportable curriculum that meets the general requirements of successful web-based courses [49] and adult learning theories [35] that could be actively distributed, easily integrated into existing curricula and used by internal medicine faculty educators. In this study, we tested whether in-person faculty development training is associated with a) use of a free web-based Alcohol Clinical Training (ACT) curriculum among physician educators, b) increased alcohol-related teaching confidence, and c) increased specific alcohol-related teaching practices.

## Methods

### The ACT curriculum

The Alcohol Clinical Training (ACT) curriculum is a federally funded, web-based curriculum created specifically for general internist educators to teach improved clinical and communication skills (screening, assessment and brief intervention) important in addressing unhealthy alcohol use in primary care settings. The ACT curriculum is based on the U.S. National Institute on Alcohol Abuse and Alcoholism (NIAAA) *Helping Patients Who Drink Too Much: A Clinician's Guide *[50]. With a special focus on health disparities, curricular topics include the spectrum of alcohol use, selected health consequences of alcohol use, epidemiology of unhealthy alcohol use, alcohol problems frequently missed, effects of physician culture on doctor/patient communication, screening, and brief intervention. The ACT curriculum was developed by and for general internists and is designed for teaching faculty, residents and medical students in a variety of teaching settings including small group conferences and large group didactic sessions. It consists of PowerPoint slides with case-based video vignettes, as well as speaker notes and audio, and learner evaluation materials. The curriculum is designed to be flexible and modifiable (i.e. slide content can be changed and videos are available as streaming or downloadable files) and can be taught using all the components together in a 3 hour workshop or by using various components separately in 45 minute sessions (i.e. preclinic conference or attending rounds).

### Pilot studies

Pilot testing was conducted to fine tune the Alcohol Clinical Training (ACT) curriculum based on input from learners in real practice settings caring for diverse (economically and culturally) patient populations. Pilot testing was performed with 3 types of physicians including residents in internal medicine, practicing community clinicians, and faculty physician educators.

#### Study design

In this controlled educational study, we analyzed baseline and 3-month follow-up survey data collected from applicants to a satellite workshop conducted at an American College of Physicians (ACP) national meeting. The study was approved by the Institutional Review Board at Boston Medical Center.

Subjects were physician educators in the U.S. who applied to attend a workshop on the use of the web-based ACT curriculum. The workshop was advertised on the ACP website and newspaper and via electronic mail to members of several medical professional organizations (e.g. Society of General Internal Medicine, Association of Program Directors in Internal Medicine). The workshop was limited to 20 participants to facilitate an interactive format and because workshop space and resources were limited. Due to this limitation, the first 20 eligible applicants were invited to attend the workshop (intervention subjects), while all eligible applicants who applied after the workshop filled were asked to enroll as control subjects (to complete assessments and have access to the curriculum website but not attend the workshop). The control group was limited by the number of additional applications received, beyond the 20 accepted for the workshop. When intervention subjects attended the workshop, control subjects were sent a letter including a description of, and web address for, the online ACT curriculum [51]. Upon completion of the study, intervention subjects received reimbursement up to $500 for travel costs or a $500 honorarium for completing the baseline assessment, attending the workshop and completing the 3-month follow-up survey; control subjects received $100 for completing the baseline assessment and 3-month follow-up survey. All subjects provided informed consent.

#### Faculty development workshop

The workshop consisted of 3 hours of in-person, interactive teaching on the effective use of the ACT curriculum, including demonstration of navigating and using website materials, modeling of teaching by expert faculty, and creating an individual action plan: a teaching project focused on using the ACT curriculum within 2 months of the workshop. Participants were required to develop an action plan objective, and to identify the target audience, setting, available resources, potential barriers, and plan for evaluation. Attendees received continuing medical education credits from Boston University.

#### Assessments

**Figure 1:**
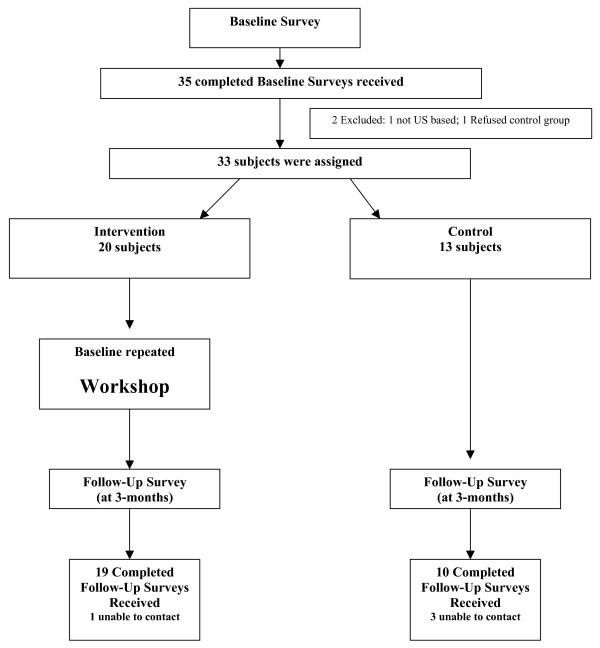
Participation Summary.

Both intervention and control subjects completed baseline surveys with their applications to attend the workshop. Follow-up surveys were mailed to all subjects 3 months after the workshop. Because up to 5 months separated completion of the baseline survey and workshop attendance, intervention subjects repeated the baseline survey directly preceding the workshop to assess whether baseline results changed (e.g. due to secular trend or in response to being selected to attend the workshop) (Figure 1: Participation Summary).

Baseline surveys included questions on respondent characteristics such as: demographics (gender, race, ethnicity, first language [English: yes/no], age, number of fluent languages other than English), residency completion year, primary teaching settings and expertise in the diagnosis and management of alcohol problems (yes/no) with any affirmative response to "Do you have expertise in the diagnosis and management of alcohol problems through: American Society of Addiction Medicine (ASAM) certification, past faculty fellowship(s), practice in an addictions specialty setting or other specified being counted as substance abuse "expertise"." Additionally, baseline surveys assessed the settings in which subjects had taught about alcohol problems (i.e., resident conferences/seminars, medical student courses or conferences, continuing medical education (CME) courses, grand rounds, morning report, inpatient attending rounds, teaching while providing clinical care, other). Note that although the term "unhealthy alcohol use" better encompasses the spectrum of use of clinical interest than the term "alcohol problems," we use the latter in describing our methods and results because it was the term in use at the time of the study (consistent with the contemporaneous NIAAA guideline) [52].

Based on work by D'Onofrio and colleagues [21], the subjects were asked at both baseline and follow-up to rate, on a 5-point Likert Scale, their teaching confidence (from "not at all" to "very") and specific teaching practices (from "rarely" to "always") in the following 5 domains: alcohol screening, assessment of readiness to change, counseling about alcohol problems, eliciting patient health beliefs, and assuring patients that they are understood [21] Follow-up surveys assessed which components (slides, notes, audio, any, none) and in which settings the ACT curriculum was used in the prior 3-months. Intervention subjects were provided a copy of their action plan and asked how much of it had been completed (none, some, or all).

#### Outcomes

The primary outcomes are baseline to follow-up change in self-reported teaching confidence (5 domains) and specific teaching practices (5 domains). Secondary outcomes included curriculum use, type of teaching settings, and frequency of alcohol-related advice sought. Degree of action plan completion was an outcome for intervention subjects only.

#### Statistical analyses

All data was analyzed with SAS/STAT software, Version 8.2 [53]. Initial analyses consisted of descriptive statistics (means, standard deviations, medians, interquartile ranges, and proportions). Comparisons were performed with 2 sample t-tests for continuous variables and chi square tests for categorical variables. Reported p-values are two-tailed, and a p-value less than 0.05 was considered statistically significant.

For the primary outcomes, we compared mean change from baseline to follow-up (calculated as follow-up score minus baseline score) in the 5 domains of teaching confidence and 5 domains of specific teaching practices both between and within groups. We also compared between-group differences in secondary outcomes. Lastly, for intervention subjects, we compared responses on the baseline application to those from the pre-workshop repeat baseline surveys.

## Results

Of the 35 physicians who completed baseline surveys, 1 was not U.S.-based and 1 refused to participate in the control group; thus 33 were enrolled (Figure 1). Of 20 intervention and 13 control subjects, 19 (95%) and 10 (77%), respectively, completed follow-up. There were no statistically significant differences in baseline characteristics by group (Table 1) including self-reported counseling and teaching others to counsel patients with alcohol problems regarding their alcohol use. One subject was missing all baseline data, with the exception of gender.

**Table 1:** Characteristics of the 33 enrolled physician educators

	**Intervention Group (N = 20)**^†^	**Control Group (N = 13)**	**p-value**
**Male (%)**	79	62	0.43
**Race (%)**			0.85
Asian	37	31	
*Black/African American*	11	8	
*White*	37	54	
*Other*	16	8	
**Hispanic (%)**	5	8	1.00
**English First Language (%)**	58	54	1.00
**Has Substance Abuse Expertise (%)**	50	54	1.00
**Mean Age**	41	45	0.14
**Mean # Fluent Languages**	2	1	0.37
**Mean # Years Since Residency**	10	11	0.56

^†^1 subject missing on all characteristics except gender

Of the 5 domains of teaching confidence and the 5 domains of specific teaching practices evaluated, compared to controls, intervention subjects increased significantly more in their confidence in teaching alcohol screening (mean change, intervention + 1.24 vs. control + 0.11, p = 0.006) and in the frequency of teaching about alcohol screening (mean change, intervention +0.56 vs. control -0.56, p = 0.02) (Table 2). Intervention subjects also increased significantly more than controls in the frequency of teaching learners to elicit patient health beliefs (mean change, intervention +0.81 vs. control -0.33, p = 0.03). Within group changes from baseline to follow-up in teaching confidence and frequency of specific teaching practices were significant for 9 of the 10 comparisons in intervention subjects, and 0 of the 10 comparisons in control subjects. The intervention subjects' pre-workshop repeat baseline surveys significantly increased compared with the baseline survey in only 1 domain – confidence in teaching to assure patients that they're understood, which did not fully explain the difference between baseline and follow-up scores (baseline to preworkshop repeat baseline mean change +0.51 vs. baseline to follow-up mean change +1.47).

**Table 2:** Baseline to follow-up change in 5 domains of teaching confidence and specific teaching practices

	Intervention (N = 18)^†^	Control (N = 9)^†^	Between- group p-value
**Teaching confidence**^**§**^			

Alcohol screening	+ 1.24**	+ 0.11	**0.006**
Assessment of readiness to change	+ 1.00**	+ 0.11	0.06
Counseling about alcohol problems	+ 1.18**	+ 0.44	0.12
Eliciting patient health beliefs	+ 1.29**	+ 0.67	0.23
Assuring patients that they are understood	+ 1.47**	+ 0.56	0.07

**Specific teaching practice frequency**^**¶**^			

Alcohol screening	+ 0.56*	- 0.56	**0.02**
Assessment of readiness to change	+ 0.44	- 0.44	0.09
Counseling about alcohol problems	+ 0.67*	- 0.22	0.08
Eliciting patient health beliefs	+ 0.81**	- 0.33	**0.03**
Assuring patients that they are understood	+ 0.94*	+ 0.11	0.18

*p < .05; **p < .01; in within-group comparisons of baseline to follow-up change

^†^Baseline data were missing for one subject with follow-up data in each group (1 of 19 in the intervention group and 1 of 10 in the control group)

^§^5-point Likert scale, where 1 = Not at all Confident and 5 = Very Confident

^¶^5-point Likert scale, where 1 = Rarely and 5 = Always

At follow-up, there was more curriculum use among intervention subjects than control subjects, though the difference was not significant (79% vs. 50%, p = 0.20) (Table 3). The most commonly used component of the curriculum was the slides, whereas use of the audio component was nearly nonexistent.

**Table 3:** Proportion with curriculum use at follow-up

	Intervention Group (N = 19) N (%)	Control Group (N = 10) N (%)	p-value
**Any curriculum use**	15 (79)	5 (50)	0.20
Slide Use	11 (58)	4 (40)	0.17
Notes Use	7 (37)	2 (20)	0.26
Audio Use	0 (0)	1 (10)	0.39
Video Use	3 (16)	1 (10)	1.00

Although not intended for this purpose, the curriculum was used for self-learning by the majority of subjects (71%) with no difference between intervention and control groups (Table 4). For teaching, the curriculum was used in a variety of settings, the most common of which were while providing clinical care (61%) and resident teaching conferences (43%).

**Table 4:** ACT curriculum teaching settings at follow-up

	**Intervention Group (N = 18^**†**^) N (%)**	**Control Group (N = 10) N (%)**	**p-value**
For my own learning	14 (78)	6 (60)	0.42
Resident teaching conferences	9 (50)	3 (30)	0.43
Medical student teaching conferences	7 (39)	1 (10)	0.19
Continuing Medical Education courses	0 (0)	0 (0)	N/A
Grand rounds	2 (11)	0 (0)	0.52
Morning report	5 (28)	1 (10)	0.37
Inpatient attending rounds	9 (50)	1 (10)	0.04*
Teaching while providing clinical care (e.g., precepting)	12 (67)	5 (50)	0.44
Other	2 (11)	0 (0)	0.52

*p < .05

^† ^Data missing for 1 subject

Among intervention subjects, 84% (16/19) completed at least part of their action plan including 8 participants who completed their entire action plan. Two of the three intervention subjects who did not complete any of their action plan also did not use any part of the curriculum.

## Discussion

In-person training for physician educators on the use of a web-based Alcohol Clinical Training (ACT) curriculum is associated with increases in confidence in teaching about alcohol screening and specific teaching practices – more frequent teaching about alcohol screening and eliciting patient health beliefs. Given the small sample size, non-significant increases are also noteworthy, including the increases associated with in-person training in confidence in teaching about assessment of readiness to change and assuring patients that they are understood, and more frequent teaching about assessment of readiness to change and counseling about alcohol problems. Also notable are the within-group findings demonstrating that intervention group confidence and teaching frequency increased significantly in 9 of 10 comparisons, which were not a result of improvements prior to the workshop. In comparison, the control group never improved significantly, and in fact, worsened in some cases. These findings suggest that in-person training of, and not only access to, this web-based curriculum can lead to improvements in alcohol-related teaching confidence and practice.

Although not significant, a greater proportion of educators who had in-person training used the curriculum. Even among those without in-person training, a substantial proportion of subjects reported using the web-based curriculum. Although physicians used the curriculum, none used the audio portion and very few used the video portion. While the curriculum was used for its intended purpose, as an education tool, surprisingly, it was most commonly used for self-study, even by educators self-selected as having an interest in teaching about alcohol.

Curricular topics for generalist physician educators are expanding in number and scope while residency duration remains the same. Considering the ineffectiveness of medical residency programs in training for alcohol screening and management [34,54], target audiences for this curriculum include both the physicians who will use the curriculum to train others, and physicians being trained. The intent of developing and making this alcohol education curriculum available is to provide faculty with a variety of educational materials (i.e. video, slides) that they can take "off the shelf," modify if desired and use in a variety of settings.

The ACT curriculum was developed by and for the same group – general internists. This approach is in keeping with the emphasis on specialty specific teaching in physicians' (adult) learning principles. This approach uses the internist teacher as a role model with credibility specifically applicable to the learner's specialty [55].

Diffusion of alcohol skills training is enhanced or impeded by fundamental characteristics of the training mechanism, such as its complexity and accessibility [56]. Many previously created alcohol curricula for physicians are less easy to access, less tailored to their audiences, and less focused. In the 1990s, NIAAA developed two multi-module curricula, which include materials available for purchase on diskette [57-59]. The Project ADEPT (*Alcohol and Drug Education for Physician Training*) curriculum is a comprehensive substance abuse curriculum for primary care physicians, which includes 7 modules, each with approximately 300 pages of instructional material. Several other non-web-based curricula are similarly lengthy, ranging from 9-hour sessions to 4-day workshops, and often address substance abuse in general, rather than being alcohol-specific, despite the fact that guidelines recommend universal screening for alcohol, but not other drugs. Many require payment or are no longer available. On the other hand, web-based materials are more easily available, and evaluations of web-based physician education have shown significant changes in both non-behavioral measures (e.g. knowledge, attitude, confidence and satisfaction) [44,45,60] as well as behavioral changes [61,62] that impact patient care [44]. With the increasing number of physicians using the Web for continuing medical education [42], it is not surprising that a number of organizations are making curricula available on the web. The NIAAA's *Helping Patients Who Drink Too Much: A Clinician's Guide*, upon which the ACT curriculum is based, is freely available on the Web and in print [50], however, it does not include the audio, video or evaluation features offered with ACT. Project Cork, Clinical Tools, Inc., the University of Florida Division of Addiction Medicine, and likely others, also provide Internet-based alcohol curricula, some of which are focused on screening and brief intervention and are geared toward physicians in general [63,64] while unlike any others, the ACT curriculum is specifically tailored to internist educators.

Several important limitations of this study evaluating the ACT curriculum should be considered. The small sample size makes it difficult to identify differences between groups and caution must be exercised when interpreting the results of non-significant findings. However, some results did reach significance. Second, the nonrandomized nature of the study could have led to confounding. For example, because enrollment in the intervention group was based on early workshop application, intervention subjects might have been more highly motivated than control subjects. However, we believe individuals in both groups were highly motivated since they all applied to attend a workshop that involved a considerable time commitment. Further, selection bias could lead to difficulty generalizing these results from this group of physician educators volunteered to travel to attend a course to a representative sample of physician educators. Nevertheless, our original intention was to study physicians with an interest in alcohol use and not to generalize beyond that population. Further, it is possible that the workshop learners considered the workshop instructors to be opinion leaders or field experts. Relatedly, subjects' specific teaching practices were not directly measured; as such it is possible that some of the findings may be attributed to social desirability bias, reporting favorable behaviors to researchers evaluating the course they attended.

Despite these limitations, these study results suggest that posting a Web-based curriculum tailored for internist educators can lead to its use, and to improvements in teaching confidence and frequency of teaching practices that are further improved when the curriculum is demonstrated in person. Furthermore, although intended for use by educators to train others, such a curriculum can be used for self-study. More sophisticated enhancements, such as audio and video components, might require more substantial faculty development efforts, and additional research is needed on both how to better disseminate these curricula, and on practice and patient-level outcomes. Nonetheless, this educational tool has the potential, perhaps in conjunction with other efforts [65,66], to improve clinical practice in an area recognized as needing substantial improvement [9].

## Conclusion

Leading professional societies recommend that alcohol screening and behavioral counseling interventions be implemented in primary care settings. But physician education to support this implementation has not been effectively or widely disseminated. This study demonstrates that a free web-based alcohol clinical training curriculum will be used by physician educators and that in-person training on the use of the curriculum can further increase teaching confidence and practices.

## Competing interests

The author(s) declare that they have no competing interests.

## Authors' contributions

DPA and RS led the creation of the study concept and design. All authors made substantial contributions to acquisition, analysis and interpretation of data and were involved in drafting and revising the manuscript for important intellectual content. All authors have read and approved the manuscript for publication.

## Pre-publication history

The pre-publication history for this paper can be accessed here:


